# Double-Lung Transplantation in a Patient with Pulmonary Type B Niemann-Pick Disease: A Valid Treatment Option

**DOI:** 10.1155/2022/5428381

**Published:** 2022-04-27

**Authors:** Víctor Manuel Mora, Joy Selene Osorio, David Iturbe, Sandra Tello, Yedra Guzmán, Laura Sánchez, José Javier Gómez, José Manuel Cifrián

**Affiliations:** ^1^Hospital Universitario Marqués de Valdecilla, Respiratory Department, Avda. Valdecilla s/n., 39008 Santander, Spain; ^2^Hospital Universitario Marqués de Valdecilla, Pathology Department, Avda. Valdecilla s/n., 39008 Santander, Spain; ^3^Hospital Universitario Marqués de Valdecilla, Thoracic Surgery Department, Avda. Valdecilla s/n., 39008 Santander, Spain

## Abstract

Niemann-Pick disease is a rare autosomal recessive disease characterized by an abnormal intracellular lipid accumulation. Type B is later in onset and a less severe form of the disease, so affected people may survive in adulthood. Storage of sphingomyelin in pulmonary macrophages can lead to interstitial lung disease. There are very few published cases of lung transplantation in patients with Niemann-Pick disease, all of them described in the last 2 years. We present here one case of a 57-year-old man successfully treated with a double-lung transplant.

## 1. Introduction

Niemann-Pick disease (NPD) is a rare autosomal recessive disease characterized by an abnormal intracellular lipid accumulation. There are three main types of NPD: types A and B are caused by a mutation in the sphingomyelin phosphodiesterase-1 (SMPD1) gene. These patients have a primary deficiency of sphingomyelinase activity that produces an intracellular lipid accumulation and large foam cells loaded with lipids in the reticuloendothelial system of the spleen, liver, lungs, bone marrow, lymph nodes, blood vessels, Schwann cells, central nervous system, and retina cells. [1]. Type A is the most serious and an early-onset form. It is characterized by hepatosplenomegaly, progressive loss of motor neurons, and death in the first years of life. Type B is later in onset and a less severe form of the disease, with a good prognosis in adulthood. It is characterized by hepatosplenomegaly, which leads to thrombocytopenia due to hypersplenism and in more severe forms, liver cirrhosis [2]. Storage of sphingomyelin in pulmonary macrophages can lead to interstitial lung disease, frequent respiratory infections, and respiratory failure, which can determine the outcome of the disease [3]. Other systemic manifestations include ocular abnormalities (macular halos and cherry red macules), and although most patients do not present neurological abnormalities, central and peripheral nervous system affection have been described [4]. The global prevalence of types A and B combined is estimated at 1 : 250,000 [5].

Type C is determined by mutations in NPC1 and NPC2 genes, which creates alterations in the cellular processing and transference of cholesterol and other lipids.

As mentioned before, NPD type B can present with severe pulmonary involvement due to interstitial lung disease and severe respiratory failure, which can lead to death. Lung transplantation has been described as a possible therapeutic option for these patients.

## 2. Case Presentation

A 57-year-old man, with a 20 pack-year smoking history, was diagnosed at the age of 10 with a possible miliary tuberculosis due to an interstitial lung pattern on a chest X-ray. He received specific tuberculosis treatment, without radiological improvement. A lung biopsy was performed in which alveoli with macrophages loaded with vacuoles were observed. Later on, he was diagnosed with hepatosplenomegaly. At the age of 47, he required a splenectomy due to spontaneous splenic rupture. A liver and a skin biopsies were performed, suggesting a deposition disease. The skin biopsy also revealed very low levels of sphingomyelinase in cutaneous fibroblast culture (6.5%), establishing the diagnosis of type B NPD. The patient had been referred from a tertiary hospital from a different administrative area. But the skin and lung biopsies, on which the diagnosis was based, had been done at another hospital from the one that referred him from another different area. For all these reasons and due to the speed of progression of the disease, it was not possible to access these samples before the transplant.

He is the father of two children. The only information we have in our hospital is that their children are carriers of SMPD1 gene mutation, but they have not developed any symptoms compatible with the disease, and they are healthy. In the same way, they are being followed in another hospital, and we do not have access to further information.

**Figure 1:**
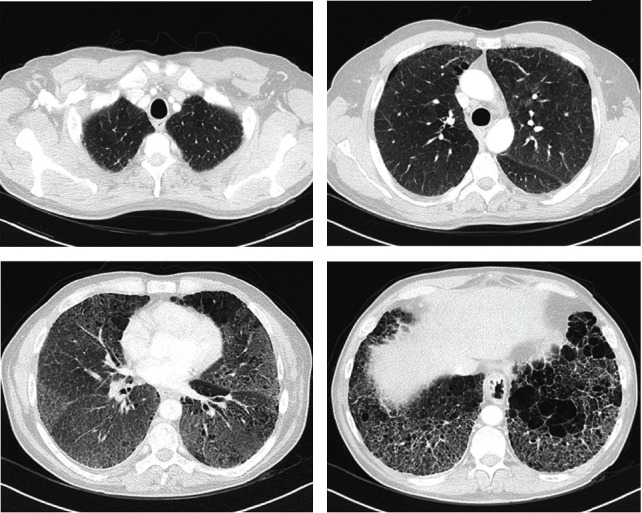
Characteristic crazy paving pattern in a patient referred to lung transplantation because of Niemann-Pick type B disease.

He had extensive pulmonary involvement at chest computed tomography (CT) with a diffuse interstitial disease and a radiological pattern of “crazy paving” in the lower and middle lung fields (Figure 1). His pretransplant pulmonary function tests demonstrated preserved lung volumes (FEV_1_: 2960 ml (95%), FVC: 4190 ml (106%), and FEV_1_/FVC: 70.70) and a significantly reduced adjusted DLCO (DLCO: 24% and DLCO/VA: 31%), his blood gas at room air showed hypoxemia (PaO_2_: 62 mmHg, PaCO_2_: 39 mmHg, and HCO_3_: 24 mmol/L), and he covered 498 meters in the 6-minute walking test, but with a desaturation up to 77% using oxygen at 3 liters/minute. He had hepatomegaly without cirrhosis, but no neurological or visual alterations. He was at functional class 4 of modified Medical Research Council (m-MRC) scale with rapidly worsening dyspnea in the last 6 months; hence, in the absence of serious extrapulmonary organ dysfunction, he was accepted as a candidate for lung transplantation.

On December 2019, the patient received a bilateral lung transplant, with no relevant surgical incidents. He was extubated 36 hours after the transplant. A right phrenic nerve paresis was observed, which did not require ventilatory support, and he did not have evidence of primary graft dysfunction. He was discharged from the intensive care unit to the hospital ward 4 days after the surgery.

At the time of awakening, the patient reported visual disturbances without clear diplopia or blurred vision. A cranial CT scan, performed 4 days after transplant, revealed “cortico-subcortical hypodense foci in posterior cortical border territories compatible with acute infarcts. Other hypodense foci were noticed in the anterior border of centrum semiovale that could correspond to acute infarcts, although difficult to assess due to its small size.” He was examined by ophthalmology, without ocular structural alterations, or motor deficits in the oculomotor muscles. The patient was also examined by neurology, without any clear pathological finding. The evaluation was completed with an electroretinogram and a visual evoked potential study, which were normal. It was concluded that the findings were related to acute ischemic lesions.

On day 24th posttransplantation, he began with sudden dyspnea, tachypnea, desaturation, central cyanosis, and plegia in the left arm with a muscular strength 0/5. An urgent chest angio-CT scan was performed in which pneumothorax and pulmonary embolism were not seen. A cranial angio-CT was also made, without any evidence of acute ischemic or embolic lesions. Later on, he began with a focal seizure with myoclonus in the left arm and then a generalized seizure, which was resolved with the administration of 10 mg of diazepam. The patient started antiepileptic treatment with levetiracetam, and the neurological symptoms subsided. A cranial magnetic resonance imaging, performed on the 28th day posttransplantation, revealed the same lesions with some hemorrhagic transformation. All of these findings were interpreted as watershed cerebral infarctions, probably secondary to hypotension during surgery.

Induction immunosuppression with basiliximab was administered (20 mg 2 hours before unclamping the first pulmonary artery and other 20 mg 96 hours after the first dose) followed by immunosuppression with tacrolimus (based on serum levels, with a target level of 12-15 mcgs/L for the first months), mycophenolate mofetil (1000 mg every 12 hours), and corticosteroids (initially in pulses of methylprednisolone, followed by 1 mg/kg/day of prednisone for the first few weeks, with a subsequent descending regimen), but with the first radiological alterations in the central nervous system and the suspicion of a posterior reversible leukoencephalopathy syndrome (PRES), a change in immunosuppression was made to cyclosporine (based on serum levels, with a target level of 350-450 mcgs/L for the first months). Although the alterations in the central nervous system were finally classified as ischemic, the same immunosuppression scheme was maintained due to a satisfactory respiratory evolution.

In the transbronchial biopsy, performed 3 weeks after the surgery, an acute cellular rejection grade A1 of the Working Formulation (WF) of International Society of Heart and Lung Transplantation (ISHLT) was diagnosed. It was decided not to treat with high doses of corticosteroids in the context of neurological complications and the frailty of the patient, and because he was respiratory asymptomatic without clinical or radiological signs of rejection. According to our protocol, grade A2 of the WF of ISHLT rejections or higher is always treated, but grade A1 rejections without symptoms or signs of rejection during the first month are not treated with high doses of corticosteroids due to the lack of evidence of their benefit effect.

**Figure 2:**
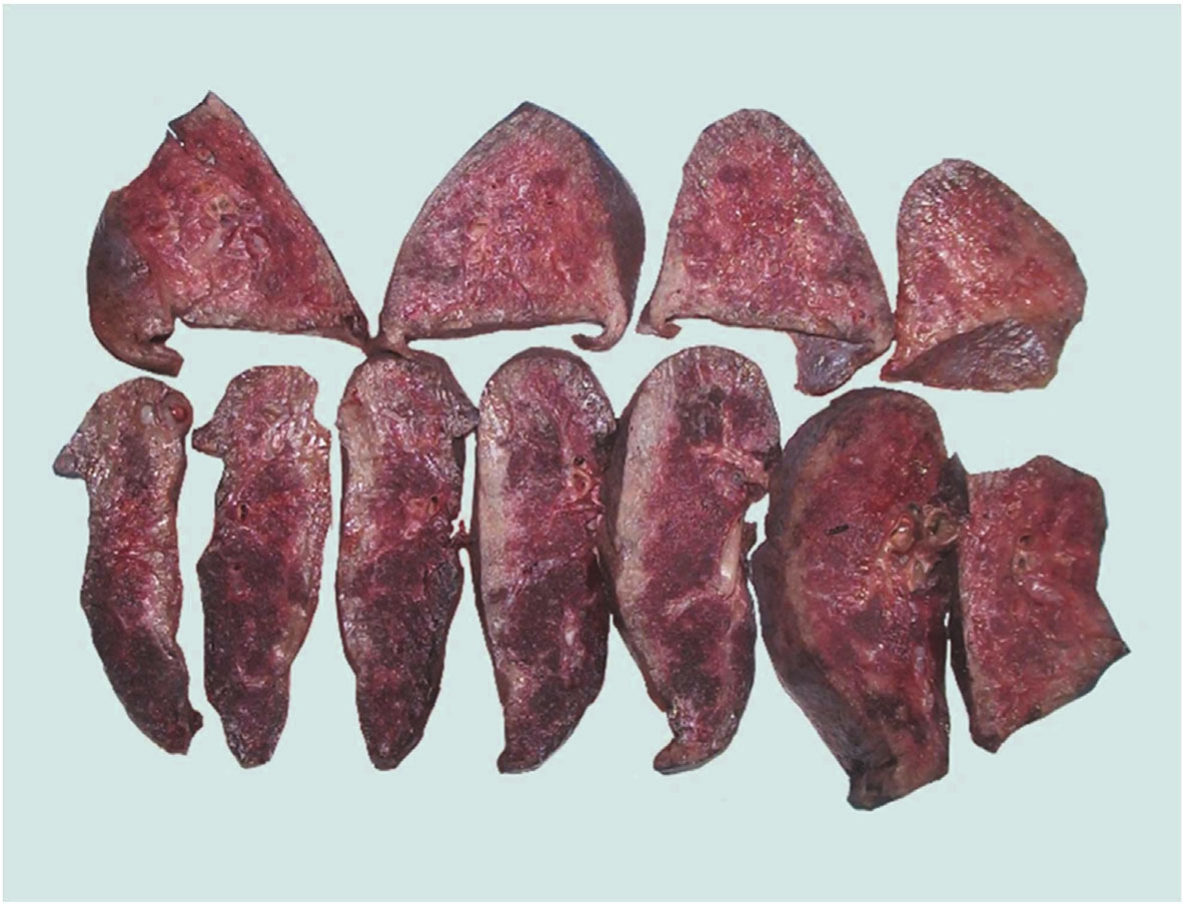
Macroscopic view of the explanted lungs.

**Figure 3:**
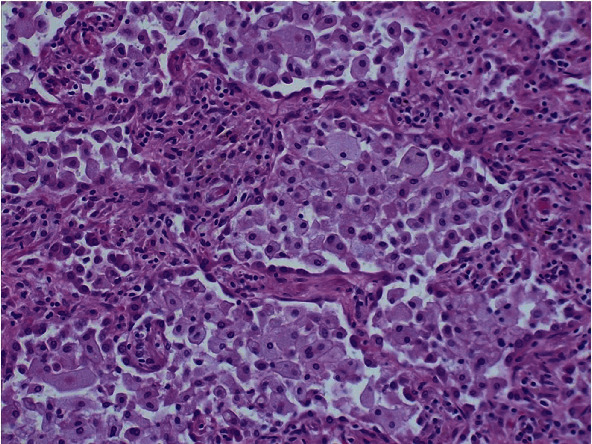
Microscopic section of lung parenchyma. Morphological pattern of unclassifiable interstitial pneumonia with foamy histiocytes suggestive of Niemann-Pick disease.

**Figure 4:**
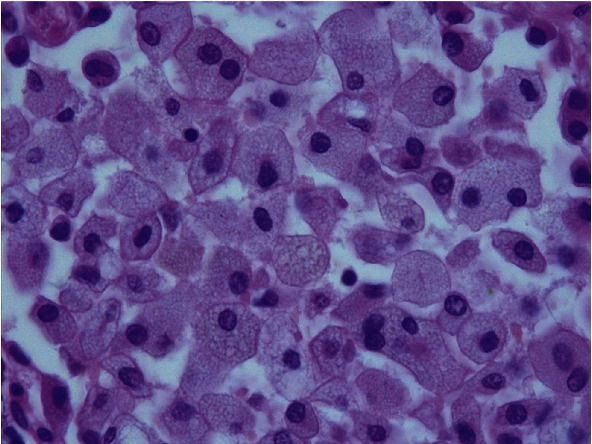
At higher magnification, foamy histiocytes.

Histopathologic findings in the explant lungs revealed a morphological pattern of unclassifiable interstitial pneumonia with foamy histiocytes suggestive of NPD (Figures 2–4). It was also found an incidental microscopic acinar adenocarcinoma pT1a, pN0 UICC Stage IA1 in the right lung, without any involvement in the 26 lymph nodes analyzed. Due to its microscopic size, without lymph node involvement, with a very early stage, no changes in immunosuppressive treatment were considered necessary.

He did not have any infectious complication, and he was discharged from the hospital 31 days after the surgery.

During follow-up visits, there was a progressive improvement in pulmonary function tests, reaching their maximum FEV_1_ values 11 months after the transplant without related complications. There was no evidence of pulmonary involvement in the CT scans of the chest, and he maintained m-MRC functional class 0.

Electrophysiological evaluation of the phrenic nerve was repeated 15 months after the transplant, and it was normal.

He remained with visual disturbances which improved a little after undergoing cataract surgery, but until this day, these disturbances have not been resolved, but without motor disturbances. The patient has been receiving levetiracetam without further seizures. He has not had abnormal liver function measured by fibroscan with 4.9 kPa, performed one-year posttransplant.

23 months posttrasplantation, the patient maintains a good quality of life, without having any relevant complications.

## 3. Discussion

NPD is a multiorgan systemic disease. There are very few published cases of lung transplantation in patients with NPD, all of them described in the last 2 years. Mannem and colleagues described in 2019 a case of a 62-year-old man, with pulmonary type B NPD, who underwent double-lung transplant and died 29 days after the surgery. He had pulmonary involvement consisting of NPD cells in the autopsy [6]. In the same year, Ding et al. described another case of a 39-year-old male who underwent liver transplant and also required a double-lung transplant at 48 years of age due to lung involvement. There was no evidence in this case of recurrence of the primary disease in the 9 transbronchial biopsies performed after the surgery. He had a good evolution of the lung and liver graft in the follow-up visits [7]. O'neill and colleagues also described the case of a 64-year-old man with type B NPD disease, a history of splenic rupture, and portal and pulmonary hypertension, who underwent double-lung transplant. He had a postoperative period with multiple and serious complications including primary graft dysfunction grade 3, vasoplegia, acute renal failure that required hemodialysis, atrial fibrillation, different infections, upper gastrointestinal bleeding, and cerebral ischemic changes in the frontal lobe [8]. On day 45 posttransplantation, he was diagnosed with acute cellular rejection and acute antibody-mediated rejection, but he could finally be discharged from the hospital 85 days after the transplant. Finally, Tirelli and colleagues described in 2021 the case of a 31-year-old woman with a rare pulmonary involvement consisting of bronchiectasis. She had a satisfactory evolution up to 23 months after the transplant, without liver complications [9].

Hence, lung transplant can be considered as a treatment option for patients with severe lung involvement and absence of extrapulmonary organ dysfunction, unless it is considered for a multiorgan transplant because there is no currently accepted treatment for the disease.

Lung transplantation has a high risk of major complications. The patients with systemic diseases have a higher risk, as evidenced in the postoperative evolution of some of the cases described before.

Complications in the central nervous system were evidenced in our case and the one described by O'Neill et al. These central nervous system alterations do not appear to be related to the disease itself, which can lead to central nervous system involvement, but rather appeared because of ongoing hypotension in the immediate postoperative period. In our case, the patient did not have similar symptoms before surgery and visual alterations appeared at the time of extubation probably related to hemodynamic instability.

In addition, the interstitial involvement can make it difficult to detect potentially malignant lesions. In our patient, an adenocarcinoma was identified in the right lung explant, but until this day, it has not recurred. Its microscopic size within the interstitial involvement probably did not allow it to be detected before transplantation.

In our case, no recurrence of the primary disease has been evidenced, although histological findings of recurrence have been described in at least one of the cases.

It does not seem that the involvement of other vital organs, such as the liver, gets worse after lung transplantation.

Therefore, lung transplantation can be considered as a treatment option in patients with NPD and advanced lung involvement.

## Data Availability

The case report data used to support the findings of this study are included within the article.

## Abbreviations

NPD: Niemann-Pick disease
SMPD1: Sphingomyelin phosphodiesterase-1
DLCO: Diffusing capacity for carbon monoxide
FEV_1_: Forced expiratory volume in one second
FVC: Forced vital capacity
CT scan: Computed tomography scan
ISHLT: International Society of Heart and Lung Transplantation
m-MRC: Modified Medical Research Council scale.
